# Wildlife Interactions on Baited Places and Waterholes in a French Area Infected by Bovine Tuberculosis

**DOI:** 10.3389/fvets.2016.00122

**Published:** 2017-01-16

**Authors:** Ariane Payne, Sixtine Philipon, Jean Hars, Barbara Dufour, Emmanuelle Gilot-Fromont

**Affiliations:** ^1^INRA, Agroecology UMR 1347, Dijon, France; ^2^French Hunting and Wildlife Agency (ONCFS), Studies and Research Department, Wildlife Disease Unit, Gières, France; ^3^Alfort National Veterinary School (ENVA), Epidemiology Unit EPIMAI, Maisons-Alfort, France; ^4^Lyon 1 University, CNRS, UMR 5558 LBBE, Villeurbanne, France; ^5^VetAgro-Sup, MIPIE, Veterinary Public Health Unit, Marcy-l’Etoile, France

**Keywords:** multi-host system, wildlife, interactions, *Mycobacterium bovis*, camera traps

## Abstract

Interactions among wildlife species are major drivers for the transmission of multi-host pathogens, such as *Mycobacterium bovis*, which also affect livestock. Although France is officially free from bovine tuberculosis (bTB), some areas are still harboring infection in cattle and wildlife. We aimed at characterizing the visits of susceptible wild species (badger, red deer, and wild boar) at baited places and waterholes, considered as possible hotspots for contacts. We described the visits in terms of frequency, duration, and number of individuals and studied the influence of the season. Then, we estimated the frequency of intraspecies and interspecies interactions occurring at baited places and waterholes which may lead to bTB transmission, including direct and indirect contacts through the soil or water. We used camera traps placed on baited places and waterholes on 13 locations monitored during 21 months. The number of visits, their duration, and the number of individuals per visit were analyzed by generalized linear mixed models for each targeted species. The frequency of the interspecies and intraspecies interactions was also analyzed separately. The season, the type of site (baited place or waterhole), and the location were the explanatory variables. Badgers’ visits and interactions were more frequent than for other species (mean: 0.60 visit/day and 5.42 interactions/day) especially on baited places. Red deer only visited waterholes. Wild boars visited most often baited places in spring–summer and waterholes in autumn–winter. They came in higher number than other species, especially on baited places. Direct interactions were uncommon. The most frequent interspecies interactions occurred between red deer and wild boar (mean: 4.02 interactions/day). Baited places and waterholes are important interfaces between the different wild species involved in the bTB multi-host system in this area. They can thus promote intraspecies and interspecies bTB transmission. Baiting ban should be carried on and management of waterholes should be considered as tool to limit the spread of bTB in wildlife.

## Introduction

When wildlife is involved in the transmission of a multi-host pathogen, controlling this pathogen may be difficult to achieve. In wildlife, host pathology and the way the different populations are connected to each other are often poorly understood. To better control multi-host pathogens in wildlife, host ecology, behavior, and local density have to be addressed as these parameters dictate to which extent an infected host brings itself or its excretions into contact with another susceptible host (1–4).

*Mycobacterium bovis* (*M. bovis*), a bacterium belonging to the *Mycobacterium tuberculosis* complex and causing bovine tuberculosis (bTB), is a multi-host pathogen, which originally infected cattle. Because bTB raises public health and economic issues, its eradication has been attempted in many countries, allowing some of them to be granted officially free (OF) by the European commission (5). However, bTB is still enzootic in cattle in some areas and is detected locally in some bTB OF countries. Several of them are also facing infection in wildlife (5–7). In the British Isles and in Central and Southern Spain, the badger (*Meles meles*) and the wild boar (*Sus scrofa*), respectively, are considered the main wild reservoirs hampering bTB eradication (6, 7). Contrary to United Kingdom and Spain, France is bTB OF since 2001. However, bTB infections have been reoccurring in domestic cattle since 2004 in several areas (8). In the same areas, cases have also been diagnosed in red deer (*Cervus elaphus*), roe deer (*Capreolus capreolus*), wild boar, badger, and fox (*Vulpes vulpes*). Except in a single area where the red deer was considered as the main reservoir, evidence is lacking to identify maintenance hosts among these species. On the basis of current knowledge, red deer, wild boar, and badger are considered as spillover hosts, whereas roe deer and fox are considered as dead-end hosts (9–13).

The Côte d’Or “*département*” (French administrative unit roughly equivalent to a county), in Eastern France, has been one of the areas with highest bTB prevalence in cattle and wildlife in France. Two *M. bovis* strains have been identified in Côte d’Or, differing in their spoligotype and by multiple locus variable number of tandem repeat profiles (SB0120, 554311456 and SB0134, 64536436, respectively). Both strains are spatially clustered both in cattle and wildlife, confirming the epidemiological link between the different hosts (12, 14). However, in such a multi-host system, it is difficult to discriminate which type of transmission (within wildlife or between wildlife and cattle) prevails. Previous studies focused on the wildlife–livestock interface in this area (13, 15). Nevertheless, the possibility of bTB maintenance within a wild-living community including several sympatric species is a pending issue. Moreover, since 2012, badgers and wild boars infected with the SB0120 spoligotype have been detected in an area where cattle are absent (Source: *Direction Départementale de la Protection des Populations de Côte d’Or*). This situation suggests that *M. bovis* may circulate and potentially be maintained among wildlife populations without any contact with cattle. To address this question, interactions patterns among the wild bTB susceptible species are required.

Previous studies showed that artificial feeding and water points may aggregate wild populations, leading to direct and indirect interactions favoring bTB transmission (16–20). Furthermore, *M. bovis* transmission through shared feed has been demonstrated both among white-tailed deer (*Odocoileus virginianus*) and between white-tailed deer and cattle (21, 22).

The objective of this study was to evaluate to which extent intraspecies and interspecies interactions within the community of bTB wild hosts occurred at baited places and natural or artificial waterholes. We aimed at determining which season and type of site (baited places versus waterholes) were the most favorable to visits from badgers, wild boars, and red deer and interactions between them. With the results, we expect to provide insights into the risk of bTB transmission within and among wildlife species on these specific sites and, as a consequence, if action measures should be targeted toward them to better control bTB in this area.

## Materials and Methods

### Study Site

**Figure 1:**
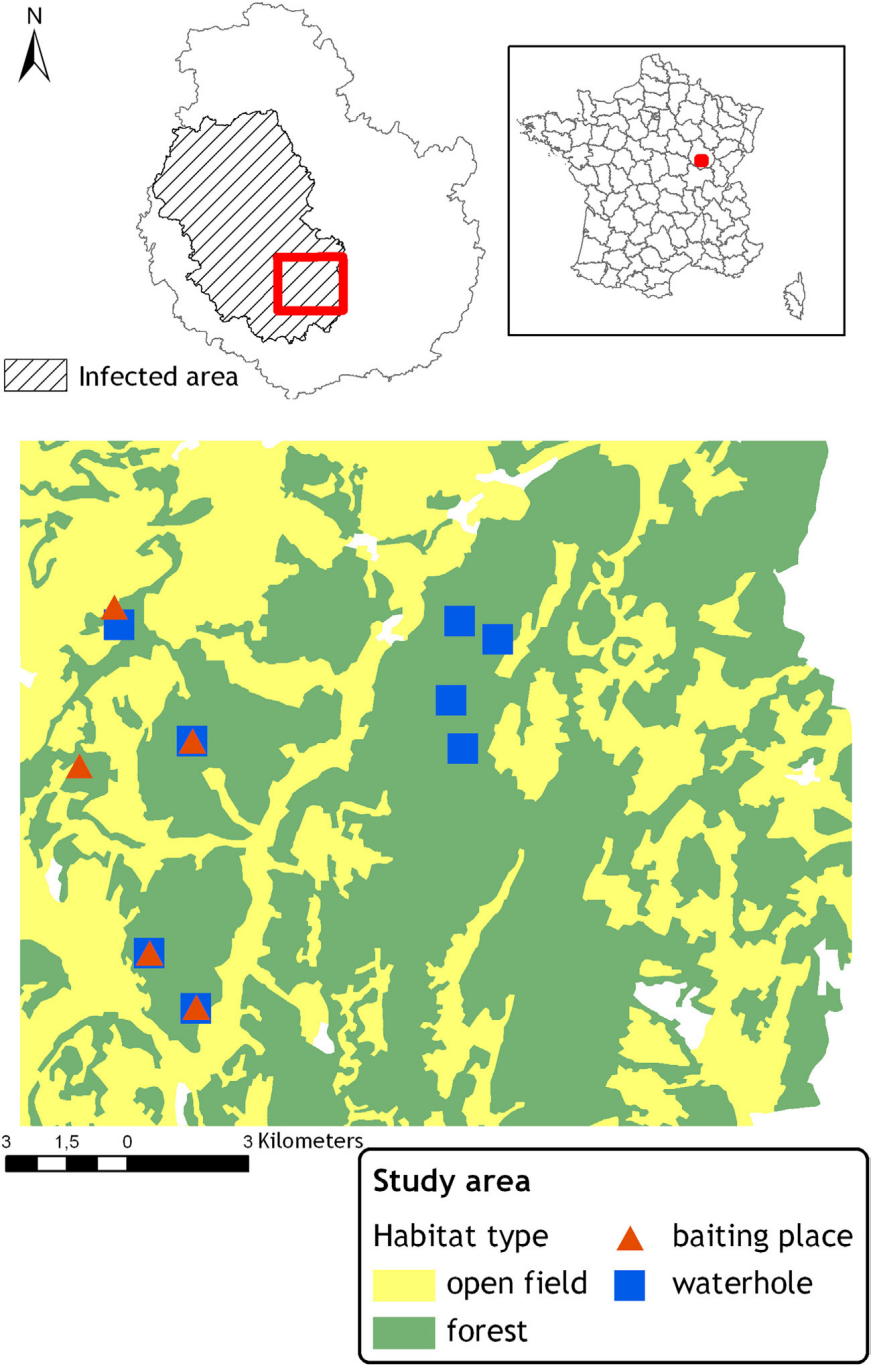
**Location of the study area in France and within the bovine tuberculosis infected area in the Côte d’Or “*département*.”** Locations of the monitored baited places and waterholes.

The study took place in the Côte d’Or *département*, where 178 cattle herds (out of around 1,700) were declared infected between 2002 and 2013 (Source: *Direction Générale de l’Alimentation, Ministry of Agriculture*) within an “infected area” of 3,000 km^2^. The study was carried out in the southern part of the infected area (Figure 1), where red deer, wild boars, and badgers coexist and had been found infected by *M. bovis*. Within this study area, bTB wildlife infection rates, estimated from culture on a pool of lymph nodes collected from hunted red deer (*N* = 655) and wild boars (*N* = 770) and trapped badgers (*N* = 275), were of 0.5% in red deer, 7.1% in wild boar, and 4.0% in badgers. Neither roe deer (*N* = 47) nor red fox (*N* = 24) was found infected in the study area (Source: *Direction Départementale de la Protection des Populations de Côte d’Or*, 13).

This study site was also chosen on the basis of a relatively homogeneous landscape (although the western part shows a more fragmented structure, see Figure 1) and species abundance. The landscape is composed of 60% of mixed forest interrupted by small valleys occupied by pastures and crop fields. In this area, the number of red deer shot per year varied from 0.4 to 1.2/km^2^ between 2001 and 2013. In wild boar, hunting bags increased from 1.3 to 3.5/km^2^ between 2005 and 2013 (13). However, the hunting effort was non-constant during the period and these evolutions may not reflect the changes in population densities. Badger density was estimated to range between 4 and 5 adults/km^2^ (13).

We selected five baited places and eight waterholes located within five different forest patches distributed in the study site, see Figure 1. Baited places were chosen among places usually baited before the ban occurring in 2011, 6 months before the beginning of the study, thus local wildlife populations were accustomed to feed on these places. Baits consisted in shelled corn, heaped on the ground, and displayed around, in a radius of about 30 m on the animals’ paths. Three kilograms of maize per place were provided and topped up when necessary, every 1, 2, or 3 days during all the study period. Waterholes consisted in small muddy ponds from 3 to 15 m long. The level of water varied according to the season. The smallest one only contained mud during the driest periods (summer). Three of them were close to a baited place also included in the protocol, see Figure 1.

### Camera Trap Survey

We used two models of infrared motion-triggered camera traps (NightTrakker NT50B, UWay Outdoors Canada, and Trophy Cam, Bushnell Outdoor Products, USA). These two camera models have similar characteristics (movement detection within a 15 m range, trigger speed set to 1 s, minimal trigger interval set to 5 s). For each baited place or waterhole monitored, a single camera was placed 150–200 cm above the ground to avoid it being damaged or moved by animals. When baited place and waterholes were close to each other, cameras were placed in order to have no overlap between the two fields of view. The cameras were programed either to take photographs or to record 20 s video footages. They worked continuously during day and night. Date and time were displayed for each photo/video.

We deployed the cameras from June 2011 to March 2013. They were rotated between the different places to monitor. We defined a session as a continuous period of monitoring on the same place with the same camera, position, and program (photo or video). All video and photos were observed for species identification and count of the number of individuals. We also noted the behavior that could be observed from the photos and the video footages. As pictures and footages were not equally informative, we remained descriptive and did not perform any analysis from behavioral data.

We defined independent visits as (1) consecutive photographs or footages of individuals of different species, (2) consecutive photographs or footages of individuals of the same species more than 30 min apart, or (3) non-consecutive photographs or footages of the different or same species (15, 23). We included the wild species that have been found infected with bTB in the study area: badger, red deer, and wild boar. We excluded visits where pictures or footages did not show any close (at a distance estimated to be more than 2 m) contact between the individuals and the baits or the waterholes, as we considered this could not lead to *M. bovis* infection or substrate contamination.

### Variables Definition

A direct interaction was registered when individuals belonging to different targeted species were seen simultaneously on the same footage or photograph. Given that the studied species are social, we considered group of individuals seen together as the epidemiological unit. As a consequence, we did not take into account direct contact occurring among them. An indirect interaction was recorded when two visits occurred within a specific time window. This time window was determined considering that a visit could entail environmental contamination lasting as long as *M. bovis* persisted in the environment. We used previous results on *M. bovis* persistence (24) that was obtained in substrates most similar to those encountered in our study (soil and water for waterholes, corn for baited places). Because the study of Fine et al. (24) was carried out in natural meteorological conditions of the Michigan, we compared the temperatures and rainfall recorded in Fine et al.’s study to those encountered in our study site, provided by a local weather station and we adapted the length of the time window considering the climatic differences between the two areas. In waterholes, we considered that *M. bovis* persistence in autumn–winter should be closest to the one found in water, while persistence in the dry period (spring–summer) should be close to persistence in soils. As a result, different time windows were chosen according to the site (baited places and waterholes) and season (30 and 15 days for waterholes in autumn–winter and spring–summer, respectively, 15 and 3 days for baited places in autumn–winter and spring–summer, respectively). We submitted our time windows assessment to a researcher working on the persistence of *M. bovis* in the environment in the same study site (Barbier, personnel communication). Table 1 summarizes the data from Fine et al.’s study, elements of comparison of climatic conditions between Michigan and Côte d’Or and the time windows used.

**Table 1:** **Choice of the time window to define indirect interaction according to the type of site and season: at each season, we compared the climatic conditions reported in Fine et al. (24) in Michigan and in our study area, considering average temperature (temp, in degree Celsius) and precipitation (rain in millimeters)**.

	Fine et al. (24)			This study		
	
Season	Temp/rain	Substrate	Persistence mean/max (days)	Temp/rain	Site	Chosen time window (days)
Autumn–winter	3.5/326.4	Soil	22/28	5.7/369.5	Waterhole (always filled with water at this period)	30
		Water	32/58		Baiting place	15
		Corn	24/37			

Spring–summer	24/492.7	Soil	8/11	15.5/465.7	Waterhole (mostly muddy at this period)	15^a^
		Water	18/48		Baiting place	3
		Corn	1.5/3			

*^a^Except for a sampled waterhole that dried during summer were we considered 10 days*.

The number of visits by badgers, red deer, and wild boar, their duration, and the number of individuals per visit as well as the number of interactions (either direct or indirect, weighted by the duration of observation) were the dependent variables. Six types of interactions were analyzed: intraspecies interactions including among badgers interactions, among red deer interactions, and among wild boars interactions and interspecies interactions including badger–red deer interactions, badger–wild boars interactions, and red–deer–wild boar interactions. The season, type of site (baited place or waterhole), and location (corresponding to the forest patch and taken as a random variable) were the explanatory variables.

### Statistical Analysis

In the first step, we described the frequency of visits for red deer, wild boar, and badgers, by computing the number of visits *per* day for each session, also with the mean and standard errors (SEs) of the number of visits *per* day. We computed the means and SEs of the duration of visits, i.e., the interval between the trigger time of the first video or photo and the time displayed at the end of the last one belonging to the same visit and the means and SEs of the number of individuals *per* visit. Animals were not individually identified, so we retained the maximum number of individuals seen simultaneously in any of the footage or photo of the visit.

In the second step, we analyzed how the number of visits from each targeted species, their duration, and the number of individuals involved varied among seasons and between waterholes and baited places. In this aim, we used generalized linear mixed models to take into account the likely dependence of visits within one forest patch. For the number of visits, a Poisson model with the location (forest patch) as the random effect was considered, as usually used to analyze count data having Poisson distribution. However, to account for data overdispersion, we considered the session as an additional random effect, leading to a Poisson-lognormal model (25). To standardize survey time among sessions, we used the logarithm of the number of surveillance days *per* session as an offset. Three separate models explaining the number of visits from badgers, red deer, and wild boar were selected. We used gamma models for the duration of visits and Poisson models for the number of individuals involved.

In the third step, we calculated the mean number and SEs of the number of the different types of interactions *per* day. Then, we analyzed how the number of interactions varied among seasons and between waterholes and baited places. Six models were built to analyze the six types of interactions as described in the previous paragraph. Details on the models are given in Data Sheet 1 in the Supplementary Material.

Model selection was performed following Zuur et al.’s (26) procedure. To select the variables to be retained in the fixed part, we started with the most complex model including all fixed effects. We considered the interaction between season and type of site because we expected the most visited sites to depend on the season according to the biology and dietary needs of the animals. We then simplified this starting model by successive steps. At each step, we fitted all possible sub-models (using the glmer function of R software) and selected the sub-model with the lowest Akaike information criterion (AIC) value. Following the parsimony principle (27), when two models had similar AIC values (difference <2), we chose the one with the fewest parameters. The significance of each variable included in the model was assessed using likelihood ratio tests (LRT). The significance of contrasts between categories was assessed using Wald tests. When models failed to converge or did not enable to correctly estimate the parameters, we merged the seasons having similar parameter values (autumn with winter and spring with summer).

As variance values were often higher than means, we checked whether overdispersion was still present in the residuals of the selected models by calculating the ratio between sum of squared Pearson residuals and degree of freedom (26). The amounts of variability explained by the fixed and random factors of Poisson models were determined using the Nakagawa and Schielzeth *R*-squared [r.squaredGLMM function of R software (28)].

Data analysis was performed using the R 2.14.1 software (29).

## Results

### Collected Data

Data were collected during 1,104 “camera days” (data collected by a given camera over a given day) distributed into 52 sessions. Due to organization issues and loss of battery power, the sessions were not homogeneous in time (mean duration ± Standard Deviation (SD): 21.23 days ± 18.45), but this was taken into account in the analyses. Baited places and waterholes were each surveyed during 27 and 25 sessions and accounted for 416 and 688 camera days, respectively (see Table S1 in the Supplementary Material). A total of 10,137 pictures and 3,416 video footages were recorded. We excluded 44.2% of them, corresponding to non-target species, i.e., roe deer, foxes, wildcats (*Felis silvestris*), *Mustelidae* other than badgers, and dogs or because it contained unreadable images (too blurry or hidden by fog). One hundred ninety-two visits were excluded because the targeted species had no close contact with baits or waterholes.

A total of 979 visits from the targeted species were recorded. Among these, badgers were seen most frequently with 443 visits (45%), followed by wild boars (368 visits, 38%) and red deer (168 visits, 17%). Among the two other bTB susceptible species excluded from the study, we observed 422 visits from roe deer and 15 visits from red foxes. Baited places received 816 visits, whereas 600 visits were observed on waterholes. Visits occurred on every monitored site.

### Frequency and Characterization of Visits for Each Species

Table 2 describes the visits from badgers, red deer, and wild boar to baited places and waterholes in terms of frequency, number of individuals per visit, and visit duration, and Table 3 shows the models selected to explain the same variables. All LRT confirmed that the variables included in the selected models had significant effects. The overdispersion of residuals was limited, ranging from 0.14 to 3.05. The models selected for the frequency of visits and number of individuals showed that the location and session (random factors, corresponding to spatiotemporal variations) accounted for a part of the variations ranging from 7 to 52%. The visits that varied most with space-time were in red deer.

**Table 2:** **Description of the visits from badgers, red deer, and wild boars on baited places (BP) and waterholes (WH): mean ± SE, range for the number of visits *per* day, duration, and number of individuals *per* visit**.

	Badger		Red deer		Wild boar	
	BP	WH	BP	WH	BP	WH
Number of visits *per* day	1.11 ± 0.20	0.05 ± 0.03	0	0.26 ± 0.10	0.30 ± 0.07	0.60 ± 0.16
	0–3.21	0–0.88		0–1.94	0–1.33	0–3.37
Visit duration (min)	16.62 ± 1.54	3.78 ± 1.75	–	6.97 ± 0.78	23.32 ± 3.40	7.59 ± 0.72
	0.5–320	0.5–27		0.5–63	0.5–138	0.5–105
Number of individuals *per* visit	1.33 ± 0.03	1.04 ± 0.04	–	2.21 ± 0.15	5.89 ± 0.69	4.92 ± 0.24
	1–6	1–2		1–15	1–21	1–18

**Table 3:** **Models selected to explain the frequency of visits, their duration, and number of individuals seen in badgers, red deer, and wild boars: for each significant explanatory variable, the table gives the modalities compared, the estimate of odds-ratio with 95% confidence interval and *P*-value of the Wald test**.

Species	Response variable	Explanatory variable and modality	OR and 95% confidence interval	*P*-value of Wald test
Badger	Frequency of visits	Site: baited place	75.33 [27.82–257.3]	<0.001
	Duration of visits	Site: baited place	11.71 [5.082–27.01]	<0.001
		Season: spring	1.416 [0.716–2.780]	0.342
		Summer	0.611 [0.444–0.842]	0.003
		Autumn	0.491 [0.313–0.772]	0.002
	No. of individuals	Site: baited place	1.510 [1.017–2.355]	0.053

Red deer (waterholes only)	Frequency of visits	No significant variable		
	Duration of visits	No significant variable		
	No. of individuals	Season: spring	0.767 [0.534–1.077]	0.137
		Summer	1.027 [0.849–1.244]	0.782
		Autumn	1.006 [0.774–1.305]	0.961

Wild boar	Frequency of visits	Site: baited place	0.164 [0.046–0.502]	0.002
		Season: spring–summer	2.671 [1.149–6.407]	0.019
		Site × season	4.675 [1.173–20.75]	0.031
	Duration of visits	Site: baited place	2.518 [1.747–3.628]	<0.001
		Season: spring	1.138 [0.726–1.784]	0.573
		Summer	0.941 [0.652–1.356]	0.743
		Autumn	0.635 [0.454–0.889]	0.008
	No. of individuals	Site: baited place	2.045 [1.729–2.414]	<0.001
		Season: spring	0.937 [0.753–1.163]	0.558
		Summer	1.670 [1.445–1.935]	<0.001
		Autumn	1.566 [1.364–1.803]	<0.001

*The reference levels are waterholes (for the variable Site) and winter or autumn–winter (for the variable Season)*.

#### Badgers

Badgers visited both baited places and waterholes with an overall mean frequency of 0.60 visits*/*day. Visits by a single animal were the most frequent, although several individuals (up to six) could be observed foraging together (Table 2). Consumption of maize and foraging were the most common behaviors observed on video footages. The models showed that the frequency of visits, their duration, and the number of badgers *per* visit were all significantly lower in waterholes than at baited places. Moreover, the duration of badgers’ visits was significantly longer in spring than in summer and autumn (Table 3). Badgers were thus observed mainly searching for food, especially in spring.

#### Red Deer

We did not observe any red deer on baited places. As a consequence, we only used sessions corresponding to waterholes surveillance in the model and excluded the type of site from the explanatory variables. Red deer visited waterholes with a mean frequency of 0.26 ± 0.10 visits*/*day. Their visits lasted almost 7 min on average and more than two individuals came generally to waterholes (Table 2), corresponding generally to females with fawns or juveniles. Video footages showed adults wallowing most of the time, while fawns were seen playing in the water. Bucks were also observed scratching the soil. The frequency and duration of visits did not vary significantly with the season, while the number of red deer *per* visit was highest in winter and was significantly higher in winter than in spring (Table 3). Thus, contrarily to badgers, red deer visited only waterholes and were most numerous in winter.

#### Wild Boar

**Figure 2:**
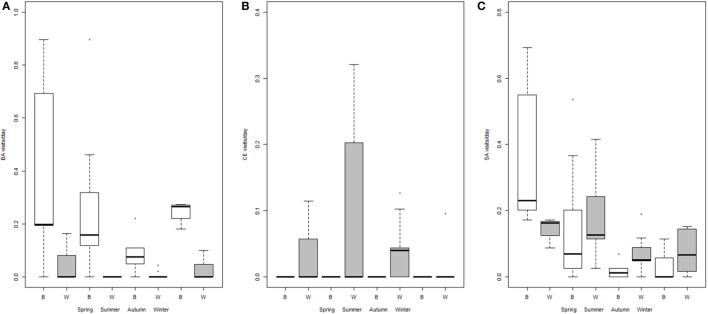
**Number of visits *per* day (log scale) for each season and type of site: baited places (B, white) and waterholes (W, gray), for badgers (A), red deer (B) and wild boar (C)**. Intervals defining boxes represent the interquartile range (IQR), while intervals out of the boxes (whiskers) show the highest and lowest values within 1.5 × IQR. Note the different scales.

Visits occurred on baited places and waterholes with an overall mean frequency of 0.44 ± 0.09 visits*/*day. Consuming maize on baited places, drinking water, and foraging were the most frequent behaviors. Wild boars were also often seen wallowing in waterholes. The visits were significantly more frequent in spring–summer, especially on waterholes (Figure 2; Table 3). Wild boars paid the longest visits on baited places among the three studied species (Table 2). Their visits on baited places lasted significantly longer than on waterholes (23 versus 8 min). The number of individuals per visit was also higher than for badgers and red deer (Table 2) and was higher on baited places than on waterholes (Table 3). In spring, wild boar made longer visits, with fewer individuals, than during other seasons (Table 3). Overall, wild boars were more frequent on waterholes, but stayed longer time and were more numerous when visited baited places.

### Frequency and Characterization of Interactions

A total of 18,132 interactions were recorded between the targeted species. Only six interspecific direct interactions were observed: three between badgers and wild boars and three between red deer and wild boars. We also observed 27 direct interactions between badgers and roe deer. All these direct interactions consisted in simultaneous presence without any nose-to-nose contact. Intraspecific interactions were more frequent than interspecific ones: 10.63 ± 2.82 and 5.22 ± 2.05 interactions *per* day on average, respectively. Interactions among badgers occurred most frequently, followed by interactions between wild boars and red deer and among wild boars (Table 4).

**Table 4:** **Number of intraspecific and interspecific interactions occurring on baited places and waterholes among badgers (B), red deer (RD), and wild boar (WB)**.

**Type of interaction**	**Intraspecific**			**Interspecific**		
	
	**10.63 ± 2.82**			**5.22±2.05**		
		
	**B–B**	**RD–RD**	**WB–WB**	**B–RD**	**B–WB**	**RD–WB**
	
Mean number of interactions *per* day ± SE	5.42 ± 2.05	1.76 ± 1.19	3.45 ± 1.27	0.14 ± 0.10	1.06 ± 0.37	4.02 ± 1.98

Table 5 shows the models selected to analyze the frequency of intraspecific and interspecific interactions. All LRT confirmed that the variables included in the selected models had significant effects.

**Table 5:** **Models selected to explain the frequency of interactions between badgers (B), red deer (RD), and wild boars (WB): for each significant explanatory variable, the table gives the modalities compared, the estimate of odds-ratio with 95% confidence interval, and *P*-value of the Wald test**.

Interaction	Explanatory variable and modality	OR and 95% confidence interval	*P*-value of Wald test
B–B	Site: baited place	1,367.2 [222.4–13,146]	<0.001

RD–RD (waterholes only)	Season: spring	170.1 [1.451–107,687]	0.034
	Summer	8.319 [0.121–1,041]	0.287
	Autumn	72.23 [2.802–10,106]	0.018

WB–WB	Site: baited place	0.065 [0.011–0.284]	<0.001
	Season: spring–summer	9.449 [2.621–41.81]	<0.001

B–RD (waterholes only)	No significant variable		

B–WB	Site: baited place	74.15 [5.218–1,053.5]	0.001

RD–WB (waterholes only)	Season: spring	539.1 [0.909–4,000,000]	0.046
	Summer	1.000 [0.034–6,484]	0.385
	Autumn	17.95 [2.533–145,353]	0.024

*The reference levels are waterholes (for the variable site) and winter or autumn–winter (for the variable season)*.

**Figure 3:**
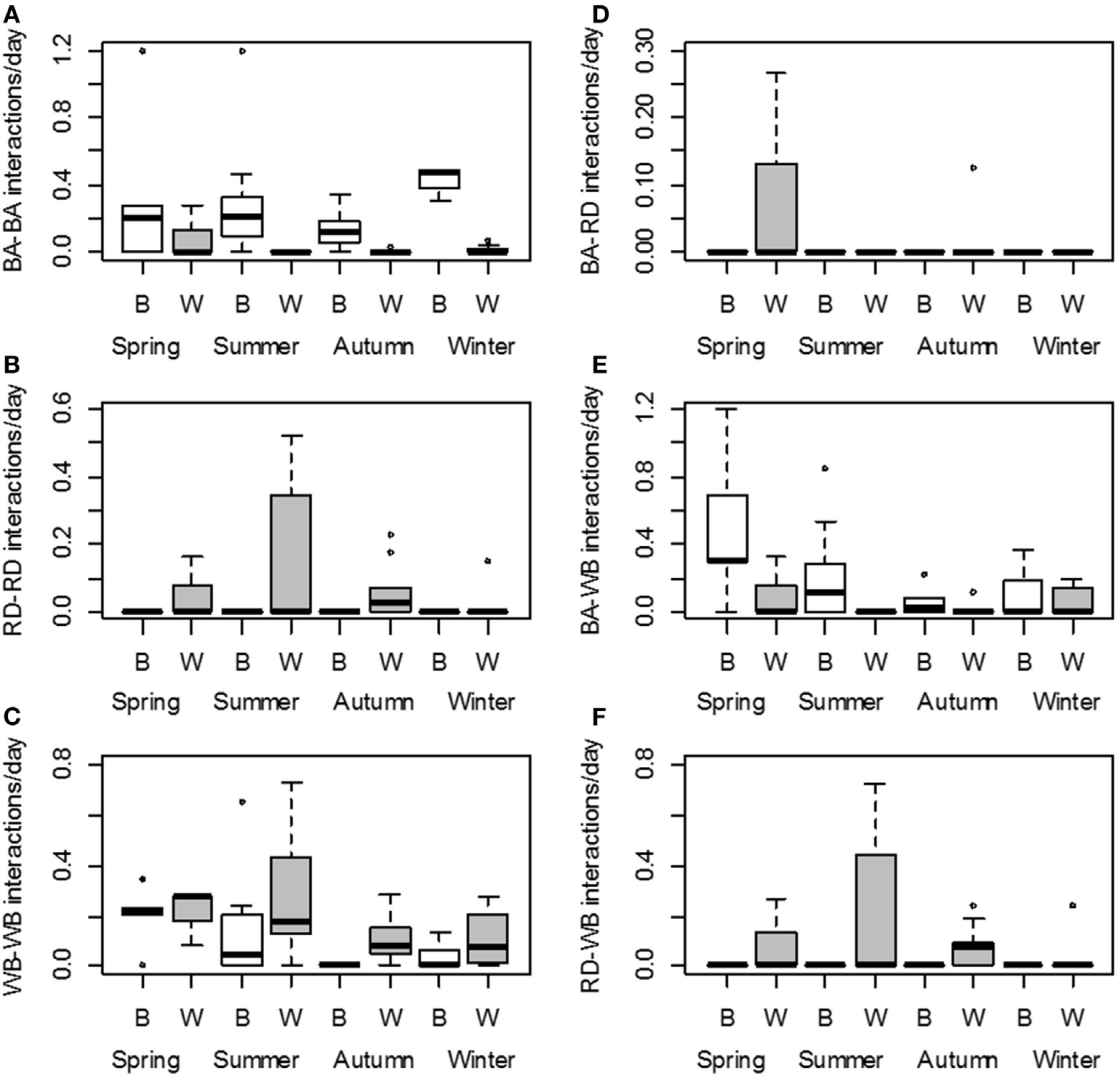
**Number of interactions *per* day (log scale) for each season and type of site: baited places (B, white) and waterholes (W, gray)**. Left panel: intraspecific interactions among badgers **(A)**, red deer **(B)**, and wild boar **(C)**. Right panel: interspecific interactions between badgers and red deer **(D)**, badgers and wild boar **(E)**, and red deer and wild boar **(F)**. Intervals defining boxes represent the interquartile range (IQR), while intervals out of the boxes (whiskers) show the highest and lowest values within 1.5 × IQR. Note the different scales.

The selected models predicted that interactions among badgers were significantly more frequent on baited places than on waterholes. Interactions among red deer occurred only on waterholes and were less frequent during winter than at other seasons. Regarding wild boars, their interactions occurred mainly at waterholes and were most frequent during spring–summer (Figure 3; Table 5).

Regarding the predicted number of the different interspecies interactions, badgers and wild boars interacted significantly more often on baited places than on waterholes. In contrast, interspecies interactions involving red deer concerned only waterholes. Interactions between red deer and wild boars were more frequent in spring and autumn than in winter (Figure 3; Table 5).

The models selected showed that the site and session (random factors, corresponding to spatiotemporal variations) accounted for 9–62%. As for the visits, the interactions that varied most with space-time were among red deer.

## Discussion

We described and compared the number and temporal pattern of visits from wild bTB susceptible species and their interactions at baited places and waterholes. Our observations showed that direct interactions between different species were uncommon and indirect interactions occurred more often within the same species than between species. Depending on the species considered, the frequency of visits, and thus the intraspecific and interspecific interactions were more frequent either on baited places or on waterholes at different seasons.

### Methods

We chose camera trapping as it is a non-invasive method, causing minimal disturbances to animals and enabling to study interactions by sampling the complete array of individuals within an area, monitor several species simultaneously, and provide behavioral insights in disease transmission scenario (30, 31). However, imperfect detection and sampling design (number, spacing, and duration of deployment) affect the interpretation of the process being sampled (32). By choosing the visit and not the individual as unit for analyses, we probably reduced the bias resulting from “false-absence” (33), i.e., when the trigger speed of the camera trap is too low to capture the animal in its field of view. The 30 min-interval we chose to define a visit was longer than the interval used in others studies carried out in Spain [15 min in Ref. (34, 35)]. This conservative choice enhanced the probability to have independence between visits.

Since individuals or groups were not identified, it was not possible to differentiate if consecutive visits (within the defined temporal window) of the same species occurred between different animals/group or between the same animal/group. As a consequence, we could have overestimated the number of intraspecies interactions.

The level and the patterns of visits and interactions between species on specific resources are highly dependent on the local density of these species and the availability of the resources (35). As a consequence, our results should not be extrapolated outside the study area or for a different study period, as these parameters are likely to differ in space and time. Moreover, the heterogeneity in the number of visits and interactions according to the location and the session, especially concerning the red deer, reveals spatial differences within our study area and temporal variations. This could be due to differences in small local scale density, abundance in resources, animals’ habits, or periodic human disturbance (such as hunting).

Because of organization issues, all locations were not surveyed evenly at all seasons (see Table S1 in the Supplementary Material). This unbalanced sampling design may have limited our capacity to detect seasonal variations.

### Occurrence and Activity Patterns of Wildlife on Baited Places and Waterholes

Badgers were more often seen at baited places than at waterholes, and made longer visits in winter and spring than in summer and autumn. Winter is a period of scarce food resources for badgers whose diet is mainly composed by earthworms, insects, fruits, and cereals (36). These results are in accordance with a previous study in the same area, where Payne et al. (15) showed that badgers visits to feeding troughs containing cereals for livestock were also more frequent than visits to water points and that winter was the most favorable season for these visits to occur. Moreover, badgers were more numerous when visiting baited places than when visiting waterholes. Several individuals (up to six), probably belonging to the same group, were sometimes observed coming and feeding together at baited places. Baited places were also places of choice for indirect interactions between badgers. As badgers were identified neither individually nor in relation to their social group, we could not conclude on whether indirect interactions were mostly within one social group or between different groups. However, these results highlight the attractiveness of baited places for this species and may consequently be considered as potential hotspots for interactions between groups.

Wild boars were most often observed on waterholes except in spring when their visits were more frequent on baited places (Figure 2). This finding is consistent with previous studies showing the attractiveness of water points for wild boars, especially under dry and hot conditions (15, 18, 20, 34, 35, 37, 38). As a result, interactions among wild boars occurred more often at waterholes than at baited places. Wild boars were seen foraging, drinking, and wallowing in water accesses. Such behaviors are common in this species to fill thermoregulation and nutritive needs (39, 40). In our study, their visits lasted longer, and involved a higher number of individuals, at baited places than on waterholes. This may be explained by the opportunistic feeding behavior of wild boars: when a resource is available and suitable, it is exploited to its maximum (41). During the study period, acorns were abundant during autumn and winter. This may be a reason why baited places were more attractive in spring–summer than in autumn–winter.

We detected red deer only on waterholes. Consistently, when estimating wildlife visits on farm facilities in the same area, Payne et al. (15) did not observe any red deer visit occurring on mangers and racks in pastures or in farm buildings, whereas visits on salt licks and water points were recorded. Hence, supplementary feeding does not seem to be attractive for red deer in this area. In Spain also, red deer were rarely observed on feeding points compared to water points or pastures (20, 34, 35). In contrast, wild deer from North America such as elks (*C. elaphus*) and white-tailed deer use cattle-feeding areas (42–44). We may hypothesize that the higher deer densities [around 10 deer/km^2^ (43)] and a scarcer food availability observed in these areas, especially in winter, explain this difference.

### Interactions and Risk of *M. bovis* Transmission

As also evidenced in previous studies, direct interspecies interactions were uncommon (15, 20, 34, 38). As a result, the risk of *M. bovis* transmission among the different studied wild species on these places should foremost be considered through indirect interactions.

Here, we defined indirect interactions as consecutive visits occurring within a specific time window compatible with *M. bovis* survival time. Our estimation was based on literature and we selected different time windows according to the meteorological conditions and the different substrates encountered in our study (Table 1). However, this estimation remains theoretical and it is difficult to assess to which extent local differences in temperature, humidity, and nature of the substrate or soil influence the local survival time of *M. bovis*. Ongoing research aiming at estimating this local survival time has been undertaken.

We found high frequencies of visits and indirect interactions on baited places and waterholes. This result suggests that these sites promote aggregation within and between wild species and may thus potentially lead to *M. bovis* transmission, as it was shown in previous studies undertaken in Spain or North America (16–19). Moreover, baited places and waterholes may play different roles in the contact network among bTB wild hosts: baited places connect rather badgers and badgers with wild boars, whereas waterholes are an important interface among wild boars, red deer, and between both species. Hence, it appears that these two types of site may be complementary in the circulation of bTB among wildlife.

To address the transmission risk, we should take into account not only the frequency of interactions but also the level of infection of the different species, their ability to excrete the pathogen, the ability of *M. bovis* to survive in the environment, and the route and the dose required to infect any other susceptible animal (45, 46). In our study area, wild boars and badgers are moderately infected and red deer display very low bTB prevalence. However, this latter species, when found infected, harbored severe, and generalized lesions, probably leading to large amount of bacilli excretion. Infected badgers may also have high capacity of shedding *M. bovis* and by different routes, whereas wild boars, showing mostly discrete and localized lesions have probably a lower ability to excrete the pathogen (13). Nevertheless, these epidemiological features may be counterbalanced by the behavioral characteristics we observed during wild boar visits since they came to baited places and waterholes in number and paid longer visits than the other studied species. Moreover, in all the studied species the main behavior we observed on pictures and video footages consisted in feeding, drinking, and wallowing on waterholes and foraging on both places. These behaviors may lead to *M. bovis* excretion or infection since oronasal route is the most common and efficient one in these species (9, 47–51). Interactions between badgers and red deer were limited (0.14/day, against 1.06–4.02 for other between-species interactions, Table 4) and between-species transmission of bTB is probably uncommon between these two species. The wild boar, by interacting with both, the red deer and the badger may act as a “bridge host” within this local bTB multi-host community as defined by Caron et al. (3).

### Implications for Management

Control strategies for bTB management in wildlife have mainly consisted in decreasing populations’ density by culling or overhunting, offal harvesting, ban of supplemental feeding (4, 52, 53). The latter measure had proven to be efficient in reducing bTB prevalence in white-tailed deer in Michigan (19, 54). It has been implemented in several high risk areas in France, including in our study area but no empirical conclusion could be drawn from this experience since other intervention tools (reducing badger, deer and wild boars densities, and offal harvesting) had been implemented concurrently or just before the baiting ban. In the light of our results, baiting should still be banned in our study site. Moreover, an evaluation of the impact of banning independently of other strategies would be helpful.

Some of the waterholes included in this study were anthropogenic and maintained by hunters to retain game on their hunting area. Limiting these artificial waterholes could thus be suggested although one could argue that it would create more aggregation on remaining water points, including those designed for livestock, which are also used by wildlife in this area (15). For instance, Cowie et al. (55) showed that reduced number of water sources are risk factor for bTB in herds, probably by forcing more animals both livestock and wildlife to visit the same locations for drinking water. Nonetheless, drying up some waterholes, especially those which were more visited and at different periods could be tried. The effects on the frequentation of surrounding water points (including those present in pastures) could be evaluated by a similar camera trapping protocol. The adaptation of water points in order to limit muddy conditions favorable to bacteria survival and to provide clean water sources has also proved relevant for other bacterial diseases (56). Compliance and cooperation of the stakeholders, hunters in this case, would be necessary to implement such a strategy.

Oral vaccinations to control bTB in wildlife are currently under development for wild boars and badgers (4). The field trials imply to census the species and the number of individuals coming to feed the baits filled with the vaccine. Our study could thus find an application by providing the frequentation of the different targeted species on baited places. Deployment of vaccinated baits on waterholes may also be considered.

## Ethics Statement

The method used in this study (camera trapping) is a non-invasive method, enabling to collect data on wild animals’ interactions without capturing, handling them, or interfering with their normal behavior.

## Author Contributions

AP, EG-F, BD, and JH designed the study. AP collected the data during the field work. SP filtered, entered, and prepared the data for the analysis. AP and EG-F performed the analysis. AP wrote the manuscript. AP, EG-F, BD, and JH participated in drafting the manuscript or revising it critically for important intellectual content. All the authors approved the submitted version of the manuscript.

## Conflict of Interest Statement

The authors declare that the research was conducted in the absence of any commercial or financial relationships that could be construed as a potential conflict of interest.
