# Supplementary material for: Wildlife Interactions on Baited Places and Waterholes in a French Area Infected by Bovine Tuberculosis
Source: Front Vet Sci. 2017 Jan 16;3:122. doi: 10.3389/fvets.2016.00122 (PMC5237639; doi:10.3389/fvets.2016.00122)
Supplement: Supplementary file 1 [file Table_1.docx]

| **LOCATION**  **(Forest patch)** | **Type of site** | **Site Id** | **Spring** | **Summer** | **Autumn** | **Winter** | **Total per site** |
| --- | --- | --- | --- | --- | --- | --- | --- |
| 1 | WH | Site 1.1 | 0 | 1 (14) | 1 (31) | 0 | 2 (45) |
|  | WH | Site 1.2 | 0 | 1 (8) | 0 | 0 | 1 (8) |
|  | WH | Site 1.3 | 0 | 0 | 1 (33) | 0 | 1 (33) |
|  | WH | Site 1.4 | 0 | 0 | 0 | 1 (24) | 1 (24) |
| 2 | BP | Site 2.1 | 2 (15) | 2 (56) | 0 | 1 (14) | 5 (85) |
|  | WH | Site 2.2 | 0 | 0 | 1 (27) | 1 (44) | 2 (71) |
|  | BP | Site 2.3 | 1 (1) | 1 (15) | 1 (31) | 1 (14) | 4 (61) |
|  | WH | Site 2.4 | 1 (15) | 2 (32) | 3 (127) | 2 (29) | 8 (203) |
| 3 | BP | Site 3.1 | 0 | 2 (16) | 1 (10) | 1 (14) | 4 (40) |
|  | WH | Site 3.2 | 1 (17) | 0 | 1 (29) | 2 (41) | 4 (87) |
| 4 | BP | Site 4.1 | 2 (16) | 1 (9) | 1 (31) | 1 (15) | 5 (71) |
|  | WH | Site 4.2 | 1 (16) | 1 (27) | 2 (120) | 2 (54) | 6 (217) |
| 5 | BP | Site 5.1 | 0 | 6 (63) | 3 (96) | 0 | 9 (159) |
| Total BP | | | 5 (32) | 12 (159) | 6 (168) | 4 (57) | 27 (416) |
| Total WH | | | 3 (48) | 5 (81) | 9 (367) | 8 (192) | 25 (688) |
| Total | | | 8 (80) | 17 (240) | 15 (535) | 12 (249) | 52 (1104) |

Supplementary Material 2

**Wildlife interactions on baited places and waterholes in a French area infected by bovine tuberculosis.**

**Authors: Ariane Payne^*^, Sixtine Philipon, Jean Hars, Barbara Dufour, Emmanuelle Gilot-Fromont.**

**Corresponding author :** ariane_payne@hotmail.com

**Supplementary table 1**: distribution of the number of sessions among the seasons, types of site (BP: baited place, WH: waterhole) and locations. The duration of the session in days are displayed in brackets.
