# Supplementary material for: Wildlife Interactions on Baited Places and Waterholes in a French Area Infected by Bovine Tuberculosis
Source: Front Vet Sci. 2017 Jan 16;3:122. doi: 10.3389/fvets.2016.00122 (PMC5237639; doi:10.3389/fvets.2016.00122)
Supplement: Supplementary file 2 [file Data_Sheet_1.doc]

***Supplementary Material 1***

**Wildlife interactions on baited places and waterholes in a French area infected by bovine tuberculosis.**

**Authors: Ariane Payne^*^, Sixtine Philipon, Jean Hars, Barbara Dufour, Emmanuelle Gilot-Fromont.**

**Corresponding author :** ariane_payne@hotmail.com

**Analysis of the frequency of visits and interactions using the Poisson-lognormal model**

Conditional on their respective means *μ_ijkl_* (proportional to the duration of session *D_ijkl_*), the number of visits or interactions *N_ijkl_* observed during session *i* on location (forest patch) *j*, during season *k* in the type of site *l* (baiting place or waterhole) follows a Poisson distribution.

*N_ijkl_* ~ P (*μ_ijkl_* x *D_ijkl_*), thus E (*N_ijkl_*) = var (*N_ijkl_*) = *μ_ijkl_* x *D_ijkl_*

With log (*μ_ijkl_*) = *α_l_* + *β_k_* + *γ_kl_ + A_j_* + *B_ijkl_*

Fixed categorical effects: *α_l_* for type of site *l*

*β_k_* for season *k*

*γ_kl_* for the interaction site x season

Random effects: *A_j_* for location, with *A_j_* ~ N (0, *σ^2^_A_*)

*B_ijkl_* for session, with *B_ijkl_* ~ N (0, *σ^2^_B_*)

Example: glmer (Nvisit ~ Site + (1|Location) + (1|Session) + offset(log(Days)), family = poisson)

**Analysis of the number of individuals per visit using the Poisson model**

Conditional on their respective means *μ_ijkl_* , the number of individuals *N_ijkl_* observed during visit *i* on location (forest patch) *j*, during season *k* in the type of site *l* (baiting place or waterhole) follows a Poisson distribution.

*N_ijkl_* ~ P (*μ_ijkl_*), thus E (*N_ijkl_*) = var (*N_ijkl_*) = *μ_ijkl_*

With log (*μ_ijkl_*) = *α_l_* + *β_k_* + *γ_kl_ + A_j_*

Fixed categorical effects: *α_l_* for type of site *l*

*β_k_* for season *k*

*γ_kl_* for the interaction site x season

Random effect: *A_j_* for location, with *A_j_* ~N (0, *σ^2^_A_*)

Example: glmer (Nindiv ~ Site + (1|Location), family = poisson)

**Analysis of the duration of visits using a gamma model**

Conditional on their respective means *μ_ijkl_* , the duration *D_ijkl_* of visit *i* on location (forest patch) *j*, during season *k* in the type of site *l* (baiting place or waterhole) follows a gamma distribution.

*D_ijkl_* ~ *Γ* (shape = *k, scale = θ_ijkl_*), thus E (*D_ijkl_*) = (*μ_ijkl_ = k x θ_ijkl_* and var (*D_ijkl_*) = *k x θ^2^_ijkl_*

With the shape parameter k considered as constant

and log (*μ_ijkl_*) = *α_l_* + *β_k_* + *γ_kl_ + A_j_*

Fixed categorical effects: *α_l_* for type of site *l*

*β_k_* for season *k*

*γ_kl_* for the interaction site x season

Random effect: *A_j_* for location, with *A_j_* ~N (0, *σ^2^_A_*)

Example: glmer (Duration ~ Site + Season + (1|Location), family = Gamma (link = log))
